# Auditory Short-Term Memory Activation during Score Reading

**DOI:** 10.1371/journal.pone.0053691

**Published:** 2013-01-11

**Authors:** Veerle L. Simoens, Mari Tervaniemi

**Affiliations:** 1 Cognitive Brain Research Unit, Cognitive Science, Institute of Behavioural Sciences, University of Helsinki, Helsinki, Finland; 2 Finnish Centre of Excellence in Interdisciplinary Music Research, Department of Music, University of Jyväskylä, Jyväskylä, Finland; 3 Department of Psychology, University of Jyväskylä, Jyväskylä, Finland; Northwestern University, United States of America

## Abstract

Performing music on the basis of reading a score requires reading ahead of what is being played in order to anticipate the necessary actions to produce the notes. Score reading thus not only involves the decoding of a visual score and the comparison to the auditory feedback, but also short-term storage of the musical information due to the delay of the auditory feedback during reading ahead. This study investigates the mechanisms of encoding of musical information in short-term memory during such a complicated procedure. There were three parts in this study. First, professional musicians participated in an electroencephalographic (EEG) experiment to study the slow wave potentials during a time interval of short-term memory storage in a situation that requires cross-modal translation and short-term storage of visual material to be compared with delayed auditory material, as it is the case in music score reading. This delayed visual-to-auditory matching task was compared with delayed visual-visual and auditory-auditory matching tasks in terms of EEG topography and voltage amplitudes. Second, an additional behavioural experiment was performed to determine which type of distractor would be the most interfering with the score reading-like task. Third, the self-reported strategies of the participants were also analyzed. All three parts of this study point towards the same conclusion according to which during music score reading, the musician most likely first translates the visual score into an auditory cue, probably starting around 700 or 1300 ms, ready for storage and delayed comparison with the auditory feedback.

## Introduction

Musical notation is a system in which visual symbols are used to represent sound patterns. The integration of visual and auditory perception is therefore essential in music reading and performance. Performing music while reading a score requires reading ahead of what is being played in order to anticipate the necessary actions to produce the notes. Score reading thus not only involves the decoding of a visual score and a comparison of it with the auditory feedback, but also short-term storage of the musical information due to the delay of the auditory feedback during reading ahead. This study investigates the mechanism of encoding of musical information in short-term memory. We aimed to distinguish between two possible mechanisms: a visual score could be kept in memory as a visual cue until the moment of comparison with subsequent auditory feedback, or, alternatively, it could be translated immediately and stored as an auditory cue, ready for comparison to subsequent auditory feedback.

In previous studies concerning a delayed symbol-to-sound matching task [1], [2], the findings were interpreted based on the assumption that visual symbols would first be translated into an auditory cue or expectation, which would then be compared to a delayed auditory input. Up to this date, we have no knowledge of actual scientific proof of this process taking place, however. On the other hand, both neuroimaging and behavioural studies have previously shown that visual cues modulate the processing of forthcoming auditory input in the context of music score reading [3]–[6]. Another recent study [7] showed that predictive visual information can modulate the processing of auditory information in a visuo-auditory matching task with very short delay. The focus of these studies, however, was on the processing after the matching rather than on the preparation before the matching. This study was thus designed to fill in that gap.

A widely accepted model of short-term memory is the three-component system proposed by Baddeley and Hitch [8], which has been expanded throughout the years. The model consisted of three components, namely, the central executive and its two slave systems, the visuo-spatial sketch pad and the phonological loop. A third slave system (episodic buffer) was added later to the model [9]. The phonological loop was described as a relatively modular system comprising a limited-capacity brief store (passive phonological store) and means of maintaining information by vocal or subvocal rehearsal (articulatory loop). The model was mainly designed for understanding language processing. In the latest update of the model (‘multi-component model’, [10]), the phonological loop has been suggested to be involved in short-term memory of music as well although the process is still not understood.

Several researchers suggested the existence of an additional tonal loop, responsible for the short-term memory of pitch [11]–[13]. This notion was supported by behavioural data in which tonal distractors caused the most interference with a delayed pitch comparison task in musically trained individuals [11], [14]. The notion was also supported by functional magnetic resonance imaging (fMRI) data, in which the phonological loop and the tonal loop showed overlapping core areas but also engaged different neural subcomponents [12]. In addition, they found that musicians recruited brain areas exclusively for the rehearsal in either of the two domains.

All previously mentioned studies were unimodal, using tonal stimuli to be compared to tonal stimuli. In our experiments, however, the tonal information had to be decoded from a visual representation *before* being compared to a subsequent sound, in order to approach a more realistic score reading situation. This approach was based on a possible cross-modal mechanism of score reading, *notational audiation*, a process in which score reading triggers auditory imagery [15]–[17]. Several researchers have suggested that audiation aids memorization of music from a score [18], [19]. However, notational audiation was most disturbed by humming a folk song while reading a different music score, more than just hearing a recording of themselves singing a folk song or tapping a rhythm while reading a different music score [16], [17]. The authors thereby concluded that notational audiation is the silent reading of musical notation involving phonatory and motor processes rather than attributing the process a sensory quality that is similar to the experience of perceiving. It is, however, not a common task of musicians to sing another melody while reading a music score. On the other hand, musicians are very used to hearing other melody lines while reading their own music score. The findings of Brodsky et al. [16] thus might reflect also exposure and training in musicians.

Studies on musical imagery did attribute a sensory quality to the process. Brain imaging studies supported this notion by showing that imagery and perception for melodies share neural structures and topographies [20], [21]. In addition, a recent fMRI study showed a quasi-automatic activation of a widespread multimodal network of brain regions (visual, auditory, audiovisual, motor, parietal, and frontal areas) in musicians while being visually presented with even a single note [22]. Schürmann et al. [23] demonstrated in a magnetoencephalographic (MEG) experiment that notational audiation induces an initial activation of left and right occipital areas, spreads to the midline parietal cortex (precuneus), and then to the left temporal auditory association areas and the left and right premotor areas. The authors stated that notational audiation includes auditory association areas that are involved in forming and recalling firmly established audiovisual associations. However, their analyses did not go beyond the first 500 ms after stimulus onset. In addition, no behavioural measures were taken to ascertain their audiation success, besides a global self-report after the experiment. Successful trials were thus not distinguished from unsuccessful ones in the analyses. This could be problematic since Huijbers et al. [24] showed different patterns of activity between successful and unsuccessful mental imagery and memory retrieval.

A delayed stimulus comparison task necessarily involves short-term memory. This type of memory is believed to be reflected by slow wave potentials. Slow wave potentials are changes in cortical polarization of the electroencephalographic (EEG) lasting from 300 ms up to several seconds [25]. They are believed to reflect depolarization of layers I and II of apical dendrites of vertically oriented pyramidal cells [26], [27]. The location of the sources of these potentials depends on task, stimulus modality, type of information processing, and motor responses involved [25], [28]. Matching words according to phonological (rhyming), semantic (category), or orthographic (upper case versus lower case) criteria results in different topographies of cortical slow-wave potentials [29]. Short-term storage of a musical score as an auditory or a visual cue may thus also result in differences in the scalp topography of slow wave potentials.

To explicitly investigate the encoding mechanism of musical information in short-term during score reading, a study was designed consisting of 1) an EEG experiment that explores how the short-term storage takes place over time when musical notes in different modality need to be compared, 2) a behavioural experiment that explores which type of distractor interferes the most with the short-term storage when a visual musical notation needs to be compared to an auditory feedback, and 3) a brief post-experimental interview of the participants about their applied strategies. In the EEG experiment, the hypothesis was that the score reading-like task would be topographically more similar to the auditory/visual task if the musical information was maintained in an auditory/visual modus before comparison. In addition, we expected differences in the evoked potential between conditions in which stimuli had to be maintained in short-term memory and conditions in which short-term memory was not needed to perform the task. In the behavioural experiment, the hypothesis was that the more similar the distractor is to the short-term storage, the graver the distracting effect would be, leading to poorer accuracy and prolongation of the reaction time.

## Methods

### 1. Ethics statement

The experiments respected the declaration of Helsinki and were approved by the ethical committee of the former Department of Psychology, University of Helsinki.

### 2. EEG experiment

#### 2.1. Participants

Fifteen male (one left-handed) professional musicians signed informed consent and participated in the EEG experiment for a small financial compensation. A follow-up study with the same participants was planned with the induction of acute stress. To rule out the hormonal influence of the menstrual cycle on the stress hormone cortisol in the acute-stress study, only men were recruited [30]. All participants were musically active at the time (practicing daily or several times per week), and none reported hearing problems or absolute pitch. Their mean age was 39.71±10.71 years (range 23–53 years). They had a mean experience (current age – starting age) of 30.33±11.71 years (range 7–47 years). The participating musicians had percussion, viola, cello, contrabass, saxophone, clarinet, trumpet, or electric guitar as their main instrument.

#### 2.2. Stimuli

The stimuli consisted of a visual (two notes on a stave) and an auditory music dyad (two sinusoidal tones). Both stimuli were presented simultaneously during 200 ms (10 ms rise and fall times). This coincides with a 1/8 note in ¾ meter in a moderate tempo (*moderato*). The 200 ms duration is also about the estimated eye fixation time during music reading for a simple score [31], [32].

**Figure 1:**
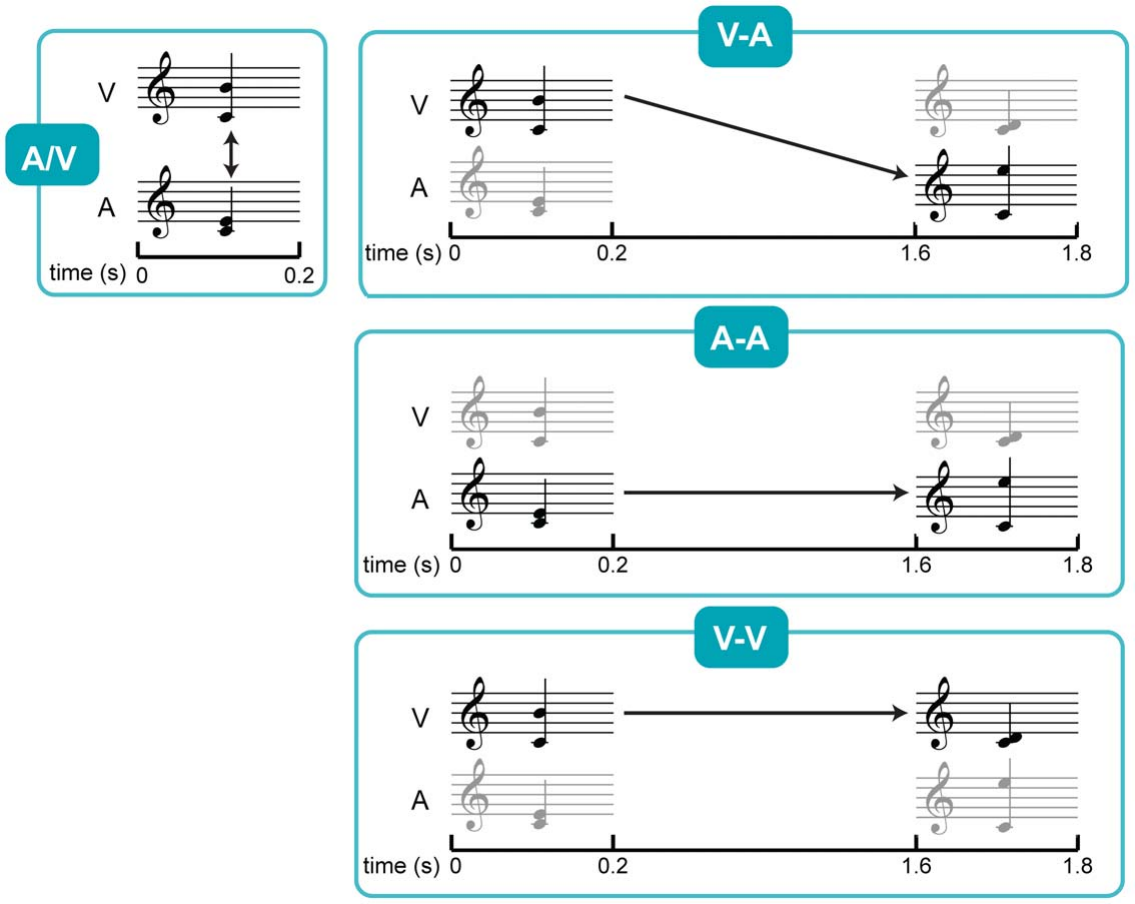
Experimental procedure of the EEG experiment. Overview of the different conditions in the EEG experiment. All stimuli pairs consisted of simultaneously presented auditory and visual stimuli, but the task differed between the conditions. A/V: simultaneously presented auditory and visual stimulus to be compared, V-A: visual stimulus of the 1^st^ pair and auditory stimulus of the 2^nd^ pair to be compared, A-A: auditory stimulus of the 1^st^ pair and auditory stimulus of the 2^nd^ pair to be compared, V-V: visual stimulus of the 1^st^ pair and visual stimulus of the 2^nd^ pair to be compared. The V-A condition was also used in the behavioural task, with and without distracters presented in the time period of 0.2–1.6 s.

The visual stimuli were displayed on a computer screen in black on a white background. The average visual stimulus, as can be seen in Fig. 1, was presented at a visual angle of 3.20°. The auditory stimuli were presented through headphones approximately 40 dB above hearing threshold, as determined by a quick assessment prior to the experiment.

All dyads were in C Major. The lower tone was always C4 (261.63 Hz) and the upper tone varied randomly between D4 (293.67 Hz) and A5 (880.00 Hz). None of the participants had absolute pitch, so the C4 served as a reference. The participants could therefore interpret the dyad as an interval or as solely the upper tone. We used pure tones, rather than harmonic tones or other spectrotemporally complex stimuli, because they do not resemble any particular instrument sounds and thus are neutral to all musicians despite the timbre of their instrument.

Stimuli were created with Finale (Makemusic, Eden Prairie, MN, USA), Adobe Illustrator CS3 (Adobe Systems Inc., San Jose, CA, U.S.A.), Adobe Audition 3.0 (Adobe Systems Inc., San Jose, CA, U.S.A.), and Matlab (The MathWorks, Natick, MA, USA). Presentation (NBS, Albany, CA, USA) was used to present the stimuli.

#### 2.3. Procedure

As in a real-life score reading situation during music performance, visual and auditory stimuli were presented simultaneously. This was done twice, at the beginning of each trial and again 1.6 s later (Fig. 1). During a following 1.4 s break, the participants had to press a button to determine whether the earlier visual stimulus was congruent with the later auditory stimulus (V-A condition). Congruent and incongruent events were randomly presented, each at 50% frequency. The visual stimulus of the first pair (the first auditory stimulus was irrelevant) was to be compared with the auditory stimulus of the second pair (the second visual stimulus was irrelevant), resembling reading ahead in a music score. The interval between the first and the second pair approached the realistic time delay while reading a score ahead (±1 s [33], [34]). This condition was then compared to three other conditions; 1) A-A condition, in which participants compared the auditory stimulus of the first pair with the one of the second pair, 2) V-V condition, in which participants compared the visual stimulus of the first pair with the one of the second pair, and 3) A/V condition, in which the simultaneously presented auditory stimulus was to be compared with the visual stimulus. It was assumed that conditions A-A and V-V would not involve cross-modal translation but did involve short-term representation within each modality. It was also assumed that condition A/V did involve cross-modal translation but did not involve short-term memory involvement. Before each block of trials, all the possible stimuli were presented in an upwards and downwards scale, in both modalities.

Prior to the experiment, the participants were given time to practice until they felt secure about their level of performance in the task. In this practice session, the participants were first given feedback on the screen about the correctness of their response. Afterwards, the participants shortly trained without the feedback as well. None of the participants required more than the maximum of 15 min. The presentation order of the different condition blocks was counter-balanced between the participants.

#### 2.4. EEG recording

The EEG recordings were performed with 128-electrode caps (Biosemi, Amsterdam, The Netherlands) with two additional electrodes, one on each mastoid. Facial electrodes were placed on the nose, the canthi (horizontal electrooculogram (EOG)), and above and below the right eye (vertical EOG). Electrode impedance was kept below 5 kΩ. Data were obtained at a 512-Hz sampling rate with an online DC filter.

The recording took place in an electrically shielded room. The participants were seated in a comfortable soft chair with head- and footrest.

#### 2.5. EEG data analysis

Analyses were performed with Matlab, using routines from the EEGLAB toolbox [35] and custom-written functions. Ocular artefacts in all data files and, in rare cases, physiological noise were removed by performing an independent component analysis (ICA). For that, data were bandpass-filtered between 0.1 and 100 Hz and periods of excessive noise or movement artefacts were removed. Independent components were then calculated by a modified InfoMax algorithm [36], as implemented in the EEGLAB function “runica”. Noise-related components were found by visual inspection of the component activations and statistical properties, and rejected. The weight matrix of the remaining independent components was then applied to 0.01–50 Hz bandpass filtered raw data. This was done because visual pruning is difficult when the data contain very low (< 0.1 Hz) frequencies. The data were referenced to linked mastoids and split into 1700 ms epochs that started 100 ms before the first stimulus pair of the trial (baseline) and ended one sample before the onset of the second stimulus pair. Epochs containing signals that exceeded ±100 µV in EEG, EOG or other additional electrodes were excluded from further analysis. Channels that showed consistently high noise levels due to suboptimal electrode contact were replaced by the average of the immediately surrounding electrodes.

Trials in which congruency occurred between the simultaneously presented stimuli of different modality in any condition except condition A/V were not included in the analysis. This was done to avoid cross-modal influences of the simultaneously presented stimuli (e.g. [37]).Only trials with correct behavioural responses were included in the analyses. Moreover, only EEG sessions in which participants responded above chance-level were included in the averaging and further statistical analysis. Finally, an additional requirement for data inclusion was a minimum of 50 epochs after artefact rejection.

To increase the signal to noise ratio, the displays of the voltage scalp maps and the comparison of the conditions V-A with V-V and A-A were very lightly smoothed with a Gaussian kernel of full-width-at-half-max of 0.6 cm. No extra smoothing filter was applied in the display or analysis of event-related potentials.

### 3. Behavioural experiment

#### 3.1. Participants

Thirty-nine professional musicians and music students participated in the behavioural experiment after written informed consent. All were musically active at the time (practicing daily or several times per week) and none reported hearing problems or absolute pitch. Their mean age was 34.16±9.55 years (range 22–53 years). They had a mean experience of 24.77±9.32 years (range 7–41 years). After an initial training session, seven participants had less than 65% correct answers and were not included in the analyses. One additional participant was excluded due to technical difficulties. Of the remaining 31 participants, 17 were classical performers (versus 14 other genres), and 22 were instrumentalists (versus 9 singers).

Twelve men had previously participated in the EEG experiment and therefore comprised a separate group (‘pre-tested’). All participants received a small financial compensation.

The three groups (Pre-tested men (*n* = 12), Novice men (*n* = 9), Novice women (*n* = 10)) did not differ in age or experience, all *p*>0.05. There was also no significant relationship between group membership and main instrument, *p*>0.05 (See Table S1 for an overview of the main characteristics of each group).

#### 3.2. Stimuli and procedure

In the behavioural experiment, the same stimuli were used as in the V-A condition of the previous EEG experiment. However, in addition to the EEG experiment, different distractors were added to the behavioural paradigm. In the distractor conditions, two distractor stimuli of the same type were presented in between the task-relevant stimuli. Participants were instructed to ignore these stimuli. There were four different types of distractors, presented in separate blocks of trails; 1) visual dyads (*DV*, two notes on a stave), 2) auditory dyads (*DA*, two sinusoidal tones), 3) spoken interval names (*IA*, auditory record of spoken interval names in Finnish, such as ‘second’), and 4) written interval names (*IV*, visual presentation of interval names in Finnish).

Noteworthy, 20% of the trials had no distractors (*NO*). The distractor stimuli had the same range and the same 200 ms duration as the stimuli in the pairs, except for the IA condition in which the duration depended on the interval name (between 200 and 420 ms). The distractors were spread semi-randomly over the 1.4 s interval (first distractor earliest 100 ms after first stimulus pair and at least 100 ms between the end of the second distractor and the beginning of the second stimulus pair). The different distractor conditions were counter-balanced in order between the participants. Each block had 30 trials and was presented twice (10 blocks in total).

After the behavioural experiment, the participants were interviewed about the strategy they used to perform the task. The first question was an open one: ‘*How would you describe your strategy?*’. The interview then went further in detail until the used strategy was clear. The musicians could use any mean to express themselves. The interviews were conducted in Finnish by a native speaker.

#### 3.3. Behavioural data analysis

The hit rate was determined as: n “congruent” responses/n congruent trials. The false alarm rate was determined as: n “congruent” responses/n incongruent trials. As a measure of sensitivity according to the signal detection theory, dprime (d′) was calculated as: z(hit rate) – z(false alarm rate), with z being the inverse normal transformation. The bias (C), as well as d′, was calculated according to Brophy [38]. Log-linear model transformations were applied to all sensitivity values [39], [40]. In addition to the signal detection theory measures, measures of the Two-High Threshold model were also reported [41], [42]. This model describes a discrimination accuracy index Pr (hit rate – false alarm rate) indicating sensitivity, and a response bias Br (false alarm rate/[1-Pr]) indicating the tendency to respond “incongruent”. The values of hit rate and false alarm rate equally underwent a log-linear transformation [42].

### 4. Statistical analysis

We compared electrical scalp potential distributions across conditions, and identified significant differences by computing paired t-tests for signals at each electrode. Correction for multiple comparisons can be achieved with e.g. Bonferroni correction by dividing the selected criterion for type 1 errors (0.05) by the number of electrodes (128). However, this correction is too conservative, because scalp potential distributions vary smoothly and electrodes in close proximity usually show highly correlated signals. We thus opted for a method that enables us to account for the empirical smoothness of the data, as opposed to an ANOVA which does not provide such tools. We estimated the smoothness of the data, as full-width-at-half-maximum (FWHM) of an equivalent Gaussian smoothing kernel, using the method described by Hagler and colleagues ([43], formula 2). We calculated the number of resolution elements [44] as an approximation of the number of independent data points by dividing the total number of electrodes by the number of electrodes contained in a circle of FWHM diameter. The number of resolution elements was then used instead of the total number of electrodes as divisor in the Bonferroni correction. Determining the number of resolution elements is usually the first step in statistical thresholding using random field theory [44]. However, this method can only be used if the width of the data is more than 3 times the width of the calculated FWHM, which is not the case in our scalp potential distributions. To locate significant differences between conditions over time, we applied the same technique to waveforms of global field potentials. Global field potentials were calculated as the root-mean-square of potentials across electrodes for each time step, referenced to the average potential. Response waveforms from all participants in different experimental conditions were compared with a paired t-test at each time step. Correction for multiple comparisons was performed by estimating the smoothness of waveforms and calculating the number of time steps equivalent to a resolution element. The length (in time steps) of a resolution element provides a “cluster-size” criterion for corrected statistical significance. Only differences between conditions that were significant for the duration of a resolution element are analyzed and interpreted.

Shapiro-Wilks tests were applied to test for normal distribution. Variables with non-normal distribution (*p*<0.05) were further analyzed with nonparametric tests as follows. Differences between the conditions in hit and false alarm rate, and sensitivity measures were tested with the Friedman test. Group differences in the various conditions were tested with Kruskal-Wallis tests. To test for differences in the behavioural experiment other than the sensitivity measures between conditions, repeated-measures ANOVA was performed with the conditions as within-subjects factor. This test was also applied for testing amplitude differences in slow wave potentials between the different EEG conditions and to verify the effect of test experience with the two male groups as between-subjects factor in the behavioural experiment. Univariate GLM models were used to test for differences in age and experience between the groups. Crosstabs with Pearson Chi square test was used to look at the distribution of the main instruments over the different groups in the behavioural experiment.

Whenever the assumption of sphericity was violated, the Greenhouse-Geisser correction for epsilon was applied. Holm-Bonferroni correction for multiple comparisons was applied where necessary. Exact uncorrected *p* values and effect size or approximations are provided for all significant tests (partial η^2^ for ANOVA, effect size for Kruskal-Wallis test: χ^2^/*N*-1, effect size for Wilcoxon test: *Z*/√*N*). For the Friedman test, Kendall's W is reported as a coefficient of concordance. All reported *p* values are two-tailed. A confidence interval of 95% was used in all tests.

## Results

### 1. EEG study

#### 1.1. Slow wave potentials

**Figure 2:**
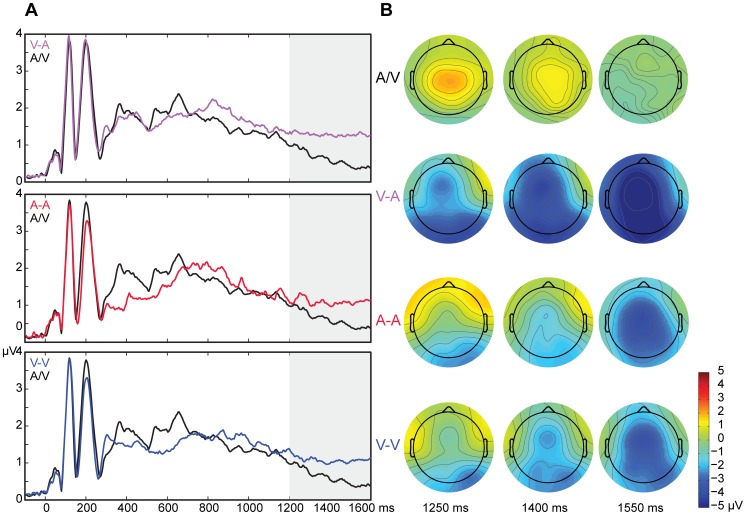
Global field potentials and voltage distributions at 1200–1600 ms. A. Global field potentials of the conditions V-A, A-A, and V-V (with short-term memory storage) compared to A/V (without short-term memory storage). The time period of 1200–1600 ms shows statistical difference in all 3 comparisons. B. Voltage maps of all conditions at time points 1250 ms, 1400 ms, and 1550 ms. There were no statistically significant differences between any of the conditions that involved short-term memory (V-A, A-A, and V-V) in whole scalp-GFP amplitude at any point of time.

To verify whether the slow wave potentials related to short-term memory were truly larger in the V-A, A-A, and V-V conditions than in the A/V condition, the integrated global field potential (GFP) between 1200 and 1600 ms was compared between the conditions. The integrated potential in a time window corresponds to the area under the curve (waveform) in that window. Integrating an event-related potential over a latency window was suggested by Ponton and coworkers [45] as advantageous compared to other measures, such as peak amplitude. There was an effect of condition on the slow wave potentials, *F*(1.79,14.31) = 7.67, *p* = 0.007, partial η^2^ = 0.49 (Fig. 2). Post hoc paired t-tests revealed that all conditions involving short-term memory processes (V-A, A-A, and V-V) had slow wave potentials of larger GFP than the condition that did not require short-term memory (A/V), with V-A: *p* = 0.005, with A-A: *p* = 0.006, with V-V: *p* = 0.007 (mean integrated GFP in µV.*ms ± SD; A/V: 143.36±40.48, V-A: 240.81±91.44, A-A: 268.55±120.71, V-V: 247.73±84.95). None of the other conditions significantly differed from each other.

**Figure 3:**
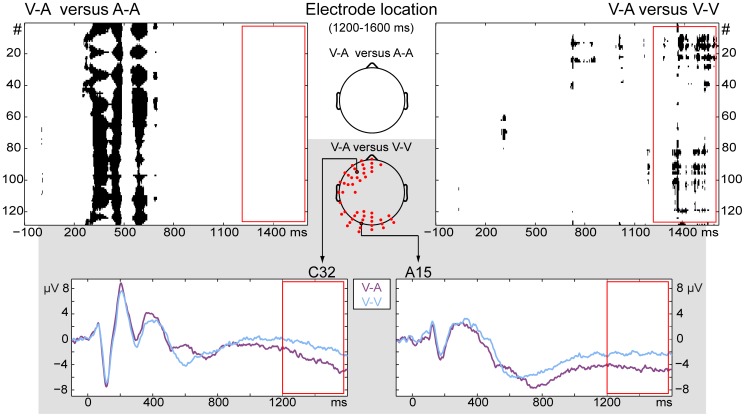
Potential differences between the cross-modal and unimodal conditions. Top row: Topographic comparison between V-A and A-A, and V-A and V-V (significant differences shown in black for all electrodes over time). During the time period 1200–1600 ms (red rectangle), V-A only showed differences with V-V. Brain responses during V-A were more negative than during Vis-Vis in that time period. Scalps in the middle show the location of the electrodes that were different during that period for at least 5% of the time. For the comparison of V-A with V-V, the differences were located in left-frontal and occipital areas. Grey rectangle: Event-related potentials of electrodes selected for display because of their central location in the two areas of significant difference.

The next goal was to explore whether the brain responses evoked during the V-A condition would correspond to or differ from the A-A or the V-V condition during the time period in which the working memory processes were expected. In the time period of 1200–1600 ms, the conditions V-A and A-A did not significantly differ from each other. Conditions V-A and V-V, on the other hand, showed left frontal and occipital electrodes to display significantly different amplitude values during that time interval. Figure 3 shows that topographical differences between the V-A and A-A are exclusively in the time window of 200–700 ms. The main topographical differences between V-A and V-V, on the other hand, are starting from 700 ms (in the occipital regions) but become most pronounced from 1300 ms onwards (in both the occipital and the left frontal regions). The whole-scalp GFP amplitude was not significantly different at any given point in time between the conditions V-A and A-A and the conditions V-A and V-V.

#### 1.2. Reaction times and correct responses

The reaction times were influenced by the condition, F(2.06, 24.77) = 8.68, p = 0.001, partial η2 = 0.42, and by congruency of the stimuli, F(1,12) = 43.95, p < 0.0005, partial η2 = 0.79 (Table RT). There was no combined effect of condition and congruency. As expected, responses in congruent trials were consistently faster than in incongruent trials throughout the conditions, p < 0.0005. The participants responded slower in the A/V condition than in the conditions A-A, p = 0.004, and V-V, p = 0.001, and V-A, p = 0.020. The conditions V-A, A-A, and V-V did not differ from each other in reaction times, all p>0.05.

There was a difference in the proportion of correct congruent responses between the conditions; *n* = 13, *df* = 3, χ^2^ = 12.05, *p* = 0.007, Kendall's W = 0.31. However, none of the pairwise comparisons reached a significant difference (all *p*>0.05). The same was true for the incongruent responses, *n* = 9, *df* = 3, χ^2^ = 12.03, *p* = 0.007, Kendall's W = 0.45; all *p* values were above significance threshold after Holm-Bonferroni correction.

### 2. Behavioural study

#### 2.1. Reaction times

**Figure 4:**
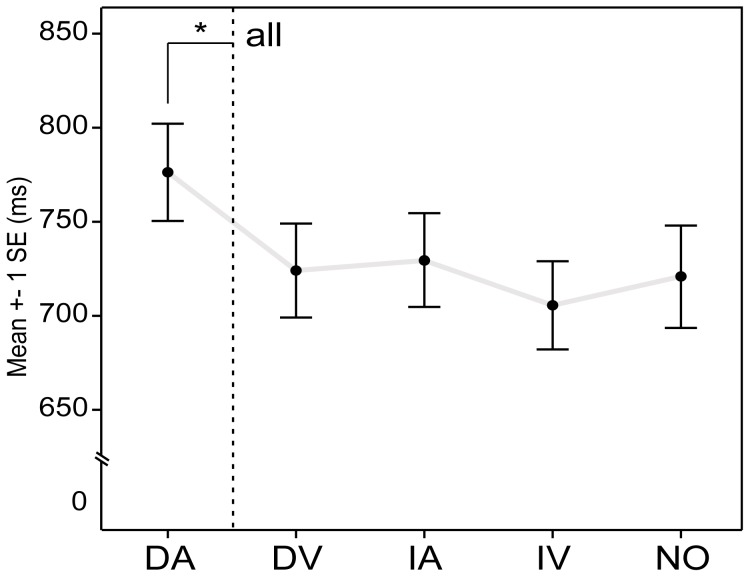
Reaction Times in behavioural experiment. Reaction times of all participants in the score-reading-like V-A condition with and without distractors. Only auditory dyads were significantly interfering the performance of the task compared to when no distracters where presented. DA: auditory dyads, DV: visual dyads, IA: auditory interval names, IV: visual interval names, NO: no distractors.

The different distractor conditions had a significant effect on the RT, *F*(4, 120) = 11.81, *p*<0.0005, partial η^2^ = 0.28 (Fig. 4, See Table S1 for all behavioural measures). Post hoc pairwise comparisons showed that only the DA condition (auditory dyads as distractors) had significantly longer RT's than any other condition, all *p*<0.05. None of the other conditions differed in RT, all *p*>0.05.

#### 2.2. Sensitivity and bias

There was an effect of distractor type on *d′*, *n* = 31, *df* = 4, χ^2^ = 9.61, *p* = 0.048, Kendall's W = 0.078. This effect was carried by the difference in *d′* between the NO and the DA condition, *n* = 31, *z* = −2.40, *p* = 0.016, *Z*/√*N* = −0.43, which was the only significant difference between the condition without distractors and any condition with distractors. The effect of distractor type on the equivalent sensitivity measure according to the Two-High Thresholds model, *Pr*, just failed to reach significance, *p* = 0.053. Neither *d′* nor *Pr* showed differences between the groups of musicians after correction for multiple comparisons, all *p*>0.05.

The bias, *C*, was also influenced by distractor type, *n* = 31, *df* = 4, χ^2^ = 10.84, *p* = 0.028, Kendall's W = 0.087. Even though the participants seemed to have responded more conservatively in the DA condition than in the others, pairwise comparisons failed to reach significance, all *p*>0.05. Similar results were found for *Br*, *n* = 31, *df* = 4, χ^2^ = 11.15, *p* = 0.025, Kendall's W = 0.090, pairwise comparisons all *p*>0.05. Again, neither *C* nor *Br* showed differences between the musician groups, all *p*>0.05.

#### 2.3. Strategies

The majority of participants of all the three groups reported adopting an auditory strategy (notes or intervals) during the behavioural experiment (Table 1). All male musicians also reported the auditory distracters as the most interfering with their performance, whereas 50% of the female musicians reported other than exclusively auditory distracters as the most disturbing or were undecided about the matter.

**Table 1:** Reports of strategy and most distracting type of distractor.

	A	V	A + V	other	missing
**Strategy (%)**					
pre-tested men	83.3	8.3	8.3	0.0	0.0
novice men	88.9	0.0	0.0	11.1	0.0
women	80.0	0.0	20.0	0.0	0.0
total	84.1	2.8	9.4	3.7	0.0
**Most distracting (%)**					
pre-tested men	66.7	8.3	8.3	0.0	16.7
novice men	66.6	22.2	0.0	0.0	11.1
women	50.0	20.0	10.0	0.0	20.0
total	61.1	16.8	6.1	0.0	15.9

*Note A: auditory, V: visual, A + V: mixed auditory and visual.*

## Discussion

An EEG experiment was designed to study the slow wave potentials during a time interval of short-term memory involvement in a situation that requires cross-modal translation and short-term storage of visual material to be compared with delayed auditory material, as it is the case in music score reading. An additional behavioural experiment was executed to determine which type of distractor would be the most interfering with the score reading-like task. The self-reported strategies of the participants were also analyzed. All three parts of this study point towards the same conclusion. During music score reading, the musician most likely first translates the visual score into an auditory cue, ready for storage and delayed comparison with the auditory feedback.

In the EEG experiment, the condition that did not require short-term storage (A/V) did indeed show smaller RMS/GFP amplitudes from the ones that did (V-A, A-A, and V-V) during a 1200–1600 ms time interval. Ruchkin et al. [46] have shown that such negative slow wave potentials reflect working memory processes rather than merely preparatory processes. This is supported by the topographical difference between the conditions that all require preparation to respond to a delayed second stimulus in our experiment.

Previous research has also shown that slow wave potentials during the retention interval of a recognition memory task varied with stimulus modality [28]. The conditions V-A and A-A only topographically differed in the 200–700 ms time window. This is the time period in which one expects topographical differences in the processing of auditory versus visual modality stimuli. The conditions V-A and V-V both start with an attended visual stimulus and thus showed no topographical differences in the 200–700 ms time window. From 700 ms onwards, however, the occipital electrodes started showing differences between these conditions and from 1300 ms onwards these electrodes were joined by the left frontal electrodes. These results are in line with previous findings of distributed activity in occipital and frontal areas, among others, during auditory imagery tasks in musicians [21], [23]. Schürmann and colleagues [23] presented a note visually and asked participants to imagine the corresponding sound. They reported imagery-specific activity in temporal, occipital, and frontal areas using MEG. Similarly to our results, auditory imaging provoked stronger activity than a simple visual task (in [23]: looking at dots) in occipital areas. Platel et al. [47] also reported activation in occipital areas (predominantly left) when participants had to pay particular attention to the pitch in a melody. The authors concluded that this could be a reflection of the participant's strategy, related to visual imagery. However, visual imagery was unlikely to be at the basis of the occipital activity found in the study by Schürmann et al. [23], nor in this study. The stronger activity found in conditions V-A and A-A compared to V-V for occipital regions has thus been found in earlier studies as well but remains mostly unexplained and requires further investigation. A second issue that remains open is why Schürmann et al. [23] reported shorter latencies than the ones found in this study. However, this is not entirely unexpected because our experiment contains a delayed-matching task and thus the translation between visual and auditory information is likely to happen later.

In the left anterior brain areas, potential amplitudes in the V-A condition (and consequently the A-A condition) were larger than in the condition V-V. This is similar to findings by Yoo and colleagues [48] who had participants imagine a single pitch with which they were familiarized before scanning in fMRI. During mental imaging, there was increased activity in inferior frontal gyri, precuneus, superior temporal gyri, anterior cingulated gyri, and middle and inferior frontal gyri. The activation was symmetrical over the hemispheres except for some areas, such as the frontal area where they found a higher activity on the left side (inferior frontal gyri). This is in accordance to our findings. In addition, Paulesu et al. [49] found increased activity in a PET study in the same left anterior brain areas as a response to reading words and non-words aloud. The authors stated that these areas are associated with word retrieval during both reading and naming tasks. It is therefore possible that reading a music score shows some topographical similarities with text reading.

Rather surprising was the finding that the condition V-A did not seem more difficult than the conditions A-A and V-V. There was no difference in reaction times or number of correct responses between these conditions. Previous research has shown that an increased memory load induces larger amplitudes of the slow wave potential [50]. There was, however, also no statistically significant difference in whole-scalp RMS/GFP amplitudes between the conditions V-A, A-A, and V-V. These findings taken together might reflect the automaticity of the score reading skills of professional musicians, even in demanding laboratory settings.

The condition A/V, however, did require longer reaction times than all the other conditions. In this condition, the simultaneously presented auditory and visual stimuli were to be compared. The participants thus had to make a cross-modal translation at that very moment, which might have delayed the response. A similar finding was found in the study by Lindström et al. [7]. In their study, reaction times were also shorter when there was a brief 300 ms delay between the visual and the auditory stimulus than when there was no delay. The authors concluded that visually induced auditory expectations speed up the processing of audiovisual associations.

The participants in our study did not make more mistakes during the A/V condition compared to the other conditions. It should be noted that this was the only condition requiring divided attention, in contrast to the conditions V-A, A-A, and V-V requiring focused attention. Larsen et al. [51] similarly found no differences in the proportion of correct responses between tasks requiring focused attention (1 modality) and divided attention (2 modalities) when auditory and visual stimuli were presented simultaneously. Their conclusion was that there must be separate pools of attentional processing resources for visual and auditory perception. The findings by Larsen et al. [51], together with the current data, could thus indirectly support the notion that an additional tonal loop exists in the multi-component model of working memory [10].

The EEG experiment pointed towards a translation from the visual to the auditory modality, probably around 700 or 1300 ms. This finding of translation before the presentation of the second stimulus is in the same line as the behavioural findings in this study. Only when distracters of the same type as the second stimulus were presented, the reactions times lengthened. The participants also most often reported auditory strategies as the one they adopted during the task. The sensitivity measures, on the other hand, were inconclusive as both methods showed contradicting results.

Even though most of the musicians reported using auditory repetition strategies to maintain the translated tones for comparison and performed well in the score reading-like task, it is impossible to ascertain that all the musicians in this study were able to imagine the tones from a visual score. The imagery in score reading is rather an audiation [15], guided by the score rather than left entirely to the imagination. According to several researchers [16], [52], this is not an ability all musicians possess. Still, as good as all classically trained professional musicians are able to read a music score with different levels of fluency. The results of this study, however, point towards a short-term memory of auditory nature during score reading. If score-based imagery or audiation would indeed be rare, there should be another cross-modal translation process involved in score reading. Further research would then be needed to unravel that process. On the other hand, when reading a score during music playing, there is the continuous auditory feedback of what is being played. This forms a basis for the audiation of the next notes to build on. Also, in this experiment the participants had the repeated presentation of C4 as a reference. Taken together that the time span of reading ahead comprises at most a few notes, the many years of solfege training of most musicians might facilitate the low-level audiation skills in those musicians.

Previous studies were not in agreement about whether reading a note produces corresponding auditory imagery or sensation in highly trained musicians [16], [17], [20]–[23]. In our results, ERPs during reading a note were indistinguishable from those during actually hearing the note. This suggests similar brain activity in both conditions, at least in so far as we access with EEG. On the basis of this result we argue that score reading evokes auditory imagery in highly trained musicians.

Although we aimed to account for many aspects of music score reading in our experiments, the current paradigm unavoidably remains a simplified version of the real-life situation. Most notably, the participants were not required to produce any movement related during score reading. Even without overt movements, trained musicians may activate premotor and/or motor cortex during score reading in order to prepare the movements to play the note. It may be speculated that this preparatory motor activity is triggered by a visual presentation of a note, rather than by an auditory presentation, because the former corresponds to actual score reading. If this is the case, then motor activity may be present in our comparison of score reading (V-A) vs. auditory only (A-A) conditions. However, brain responses in these conditions were virtually identical and we conclude that our experimental procedures either did not induce preparatory motor activity in the participants, or to such a small degree that the activity was not readily detectable with EEG.

A similar argument applies to the possibility that the visual-only condition (V-V) evokes the same degree of notational audiation as the score reading condition (V-A). If this had been the case, then brain responses in these two conditions would be indistinguishable. However, we found that they diverge from 700 ms onwards and thus conclude that notational audiation did not occur during the visual-only condition (V-V) to the same degree as during the score reading condition.

In conclusion, our results indicate that musicians, when reading a score, store the musical information in the auditory modality, ready for comparison to the delayed auditory feedback during instrument playing or singing.

## Supporting Information

Table S1 Participant characteristics, reaction times, and sensitivity measures of the behavioural experiment.
(DOC)
