# Supplementary material for: Auditory Short-Term Memory Activation during Score Reading
Source: PLoS One. 2013 Jan 11;8(1):e53691. doi: 10.1371/journal.pone.0053691 (PMC3543329; doi:10.1371/journal.pone.0053691)
Supplement: Table S1 — Participant characteristics, reaction times, and sensitivity measures of the behavioural experiment. (DOC) [file pone.0053691.s001.doc]

(Supporting material)

**Table**. Participant characteristics, reaction times and sensitivity measures of the behavioural experiment

|  | **Pre-tested men** | **Novice men** | **Novice women** | **Total** |
| --- | --- | --- | --- | --- |
| ***n*** | 12 | 9 | 10 | 31 |
| **Age** |  | | | |
| Mean (years ± *SD*) | 37.67 ± 10.05 | 33.00 ± 10.26 | 31.00 ± 7.63 | 34.16 ± 9.55 |
| Range (years) | 23 - 51 | 24 - 53 | 22 - 44 | 22 - 53 |
| **Experience** |  | | | |
| Mean (years ± *SD*) | 26.92 ± 11.02 | 21.44 ± 9.08 | 25.20 ± 7.10 | 24.77 ± 9.32 |
| Range (years) | 7 - 41 | 13 - 38 | 15 - 38 | 7 - 41 |
| **Main instrument** (*n*) |  | | | |
| Pluck | 2 | 1 | 0 | 3 |
| String | 2 | 1 | 4 | 7 |
| Percussion | 3 | 2 | 0 | 5 |
| Wood | 1 | 1 | 1 | 3 |
| Brass | 2 | 1 | 1 | 4 |
| Voice | 2 | 3 | 4 | 9 |
| **Reaction Time**  (mean ± *SD*) |  | | | |
| DA | 706.77 ± 70.62 | 824.34 ± 141.01 | 816.48 ± 72.62 | 776.29 ± 143.95 |
| DV | 640.50 ± 139.92 | 779.61 ± 135.12 | 774.42 ± 94.78 | 724.08 ± 138.94 |
| IA | 664.90 ± 153.71 | 782.83 ± 152.94 | 759.49 ± 71.33 | 729.65 ± 138.74 |
| IV | 630.50 ± 140.85 | 729.40 ± 109.28 | 774.22 ± 92.99 | 705.57 ± 130.57 |
| NO | 637.38 ± 161.07 | 747.67 ± 148.44 | 796.73 ± 94.57 | 720.80 ± 151.64 |
| **dPrime d’ (SDT)** |  | | | |
| DA | 2.45 ± 1.04 | 1.79 ± 0.85 | 2.14 ± 1.05 | 2.16 ± 1.00 |
| DV | 2.91 ± 1.20 | 1.60 ± 0.96 | 2.18 ± 0.86 | 2.30 ± 1.14 |
| IA | 2.68 ± 1.05 | 2.28 ± 0.71 | 2.31 ± 0.77 | 2.43 ± 0.87 |
| IV | 2.84 ± 0.92 | 2.06 ± 0.93 | 2.10 ± 0.89 | 2.37 ± 0.96 |
| NO | 3.07 ± 1.01 | 1.88 ± 0.94 | 2.65 ± 0.84 | 2.59 ± 1.03 |
| **Bias C (SDT)** |  | | | |
| DA | 0.049 ± 0.078 | 0.053 ± 0.053 | 0.074 ± 0.103 | 0.058 ± 0.079 |
| DV | 0.012 ± 0.029 | 0.045 ± 0.061 | 0.069 ± 0.148 | 0.040 ± 0.092 |
| IA | 0.032 ± 0.065 | 0.013 ± 0.026 | 0.048 ± 0.078 | 0.032 ± 0.061 |
| IV | 0.017 ± 0.059 | 0.000 ± 0.000 | 0.052 ± 0.081 | 0.023 ± 0.061 |
| NO | 0.027 ± 0.065 | 0.006 ± 0.018 | 0.085 ± 0.135 | 0.040 ± 0.090 |
| **Discrimination Accuracy index Pr (2HT)** |  | | | |
| DA | 0.73 ± 0.21 | 0.59 ± 0.23 | 0.66 ± 0.23 | 0.67 ± 0.22 |
| DV | 0.80 ± 0.20 | 0.53 ± 0.28 | 0.69 ± 0.17 | 0.68 ± 0.24 |
| IA | 0.77 ± 0.21 | 0.71 ± 0.15 | 0.72 ± 0.17 | 0.73 ± 0.18 |
| IV | 0.81 ± 0.15 | 0.66 ± 0.18 | 0.66 ± 0.23 | 0.72 ± 0.20 |
| NO | 0.83 ± 0.17 | 0.61 ± 0.26 | 0.78 ± 0.16 | 0.75 ± 0.21 |
| **Bias Br (2HT)** |  | | | |
| DA | 0.45 ± 0.095 | 0.46 ± 0.046 | 0.45 ± 0.072 | 0.45 ± 0.074 |
| DV | 0.49 ± 0.026 | 0.47 ± 0.037 | 0.46 ± 0.082 | 0.47 ± 0.053 |
| IA | 0.47 ± 0.077 | 0.49 ± 0.022 | 0.47 ± 0.047 | 0.47 ± 0.055 |
| IV | 0.48 ± 0.072 | 0.50 ± 0.000 | 0.47 ± 0.055 | 0.48 ± 0.055 |
| NO | 0.47 ± 0.078 | 0.50 ± 0.014 | 0.42 ± 0.137 | 0.46 ± 0.094 |

*Note. Experience: Current age – starting age, SDT: signal detection theory, 2HT: two high-thresholds model.*
